# Advances and Challenges in Metatranscriptomic Analysis

**DOI:** 10.3389/fgene.2019.00904

**Published:** 2019-09-25

**Authors:** Migun Shakya, Chien-Chi Lo, Patrick S. G. Chain

**Affiliations:** Bioscience Division, Los Alamos National Laboratory, Los Alamos, NM, United States

**Keywords:** RNASeq, microbiome, workflows, gene expression, omics

## Abstract

Sequencing-based analyses of microbiomes have traditionally focused on addressing the question of community membership and profiling taxonomic abundance through amplicon sequencing of 16 rRNA genes. More recently, shotgun metagenomics, which involves the random sequencing of all genomic content of a microbiome, has dominated this arena due to advancements in sequencing technology throughput and capability to profile genes as well as microbiome membership. While these methods have revealed a great number of insights into a wide variety of microbiomes, both of these approaches only describe the presence of organisms or genes, and not whether they are active members of the microbiome. To obtain deeper insights into how a microbial community responds over time to their changing environmental conditions, microbiome scientists are beginning to employ large-scale metatranscriptomics approaches. Here, we present a comprehensive review on computational metatranscriptomics approaches to study microbial community transcriptomes. We review the major advancements in this burgeoning field, compare strengths and weaknesses to other microbiome analysis methods, list available tools and workflows, and describe use cases and limitations of this method. We envision that this field will continue to grow exponentially, as will the scope of projects (e.g. longitudinal studies of community transcriptional responses to perturbations over time) and the resulting data. This review will provide a list of options for computational analysis of these data and will highlight areas in need of development.

## Introduction

The past few decades have seen significant advancements in sequencing technologies that have transformed how we conduct biological experiments, particularly when it comes to the study of complex microbiomes. However, most of the high throughput sequencing has focused on DNA sequencing of entire communities using either targeted approaches like PCR-amplicon sequencing of 16S rRNA genes (or other marker genes) or shotgun sequencing of all available DNA from the sample (metagenomics).

These methods have contributed to many discoveries in the past decade, helping to better characterize microbiomes from environments ranging from the human gut (Qin et al., 2010) to soil (Rondon et al., 2000) to oceans (Venter et al., 2004). Although 16S studies only directly characterize the taxonomic profile of a microbiome, it is a cost-effective option to exhaustively capture biodiversity (measuring the maximal dynamic range of relative abundance) of many samples using minimal sequencing. However, more and more studies are now using shotgun metagenomics as the advancements in sequencing technologies allow the comprehensive capture of most microbiome members while at the same time elucidating potential genes and functional pathways. One of the main limitations of shotgun metagenomics is that it does not distinguish the active from inactive members of a microbiome, and thus cannot help discriminate those that are contributing to observed ecosystem behavior from those that are merely present, presumably awaiting more favorable conditions.

Using RNA sequencing (RNASeq) to record expressed transcripts within a microbiome at a given point in time under a set of environmental conditions provides a closer look at active members. Recent advancements in mass spectrometry methods applied towards proteomics is also able to provide insight into actively expressed proteins, but is best paired with known reference genomes or a reference metagenome from which expected peptide masses can be matched. With RNASeq, relatively lowly expressed genes including the entire metatranscriptome that include non-coding RNAs can be detected, annotated, and mapped to metabolic pathways.

Biologists have long measured RNAs using targeted approaches like qPCR to quantify expression of known genes of interest. Before the advent of high throughput sequencing, microarray technologies were also widely used to measure the expression levels of known transcripts from organisms or even communities (Parro et al., 2007). With the application of next-generation sequencing (NGS) technologies to RNA, it is now possible to not only measure known transcript targets but also discover previously unknown transcripts and transcript variants directly from the sequence data.

**Figure 1:**
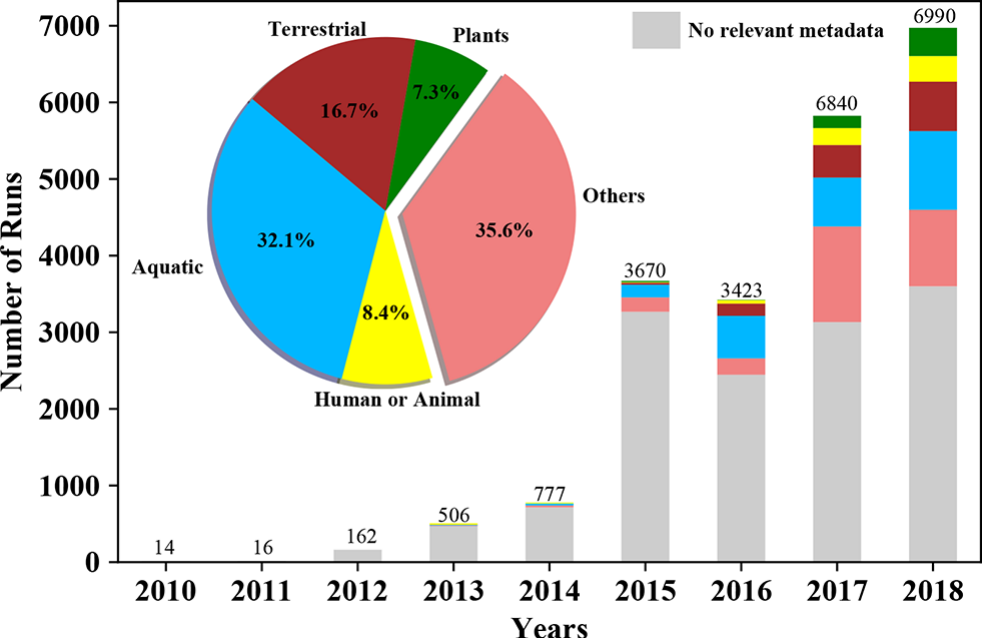
Growth of metatranscriptomics projects in public repositories, together with associated metadata, over time. Bars plots represent number of metatranscriptomic datasets (i.e. ”runs”) deposited in the NCBI Sequence Read Archive (SRA) on a per annual basis. The pie chart and the stacked bars are colored based on the source/environment (isolation_source) the sample has been isolated from. The lowest bar in grey represents the number of samples in SRA without this pertinent metadata.

In the short time since it was first introduced in the early 2000s, the number of metatranscriptomics projects, or the sequencing of RNAs from microbial communities has increased significantly (**Figure 1**). In terms of applications, the technique has been used to characterize active microbes in a community (Bashiardes et al., 2016), discover novel microbial interactions (Bikel et al., 2015), detect regulatory antisense RNA (Bao et al., 2015), and track expression of genes and determine the relationship between viruses and their host (Moniruzzaman et al., 2017). This revolutionary method is not a complete panacea however, and comes with its own set of drawbacks. As with most transcriptomic methods, experimental design is critical, sample collection is destructive and sufficient material for sequencing (or coupled experiments) is required. In addition, metatranscriptomics is not always able to capture the entire metatranscriptome due in part to the complexity (high diversity and relative ratios of members) of some microbial communities, the large dynamic range of transcript expression, the short half-life of RNA, and a number of technology-specific limitations.

In this review, we report the state of metatranscriptomics by discussing several microbiome studies from different ecosystems. We will discuss both novel findings made possible by this methodology as well as some of the shortcomings. We also list several of the available tools and workflows that have been adopted for or have been specifically designed to analyze metatranscriptomic datasets.

## Application of Metatranscriptomics Across Ecosystems

Metatranscriptomics has been applied to a number of different types of samples, from the study of human (and other animal) microbiomes, to those found in or on plants, within soils, and in aquatic environments. In this section we provide some examples of the impact metatranscriptomics has had in different fields of study.

### Aquatic Environments

One of the first metatranscriptomic studies was conducted on freshwater bacterioplankton communities (Poretsky et al., 2005), which described a total of 400 environmental transcripts from two sites. At the time, the scale of the study was dictated by the available sequencing technologies that limited the sensitivity of the method to only a few hundred genes. With the advent in the high throughput sequencing technologies, other studies on marine systems produced hundreds of thousands of reads per sample (Frias-Lopez et al., 2008; Gilbert et al., 2008) and made it possible to use metatranscriptomics to characterize the dynamics of cyanobacterial blooms in the Baltic sea (Berg et al., 2018), the detection of small RNAs in the open ocean (Shi et al., 2009), and resolve viral-host relationships of marine eukaryotes (Moniruzzaman et al., 2017).

### Terrestrial Environments

Soils are some of the most diverse ecosystems in the world. They typically harbor incredible numbers and a broad diversity of eukaryotes, archaea, bacteria, and viruses. These complex microbiomes are frequently characterized using metagenomic sequencing, but only a few of studies have performed metatranscriptomics to decipher active microbes from more sedentary soil residents. For example, in a recent study to identify functionally active organisms in soil microbial communities, metatranscriptomes revealed that *Verrucomicrobia,* which are regularly found in high abundance in soils, were not as highly active as their abundance would otherwise suggest (White et al., 2016). Upon further analyses, authors showed that the high abundance of *Verrucomicrobia* at DNA level was partly due to presence of metabolically inactive organisms. Since it is possible to sequester eukaryotic mRNA during sample preparation (e.g. using polyA tail hybridization), metatranscriptomics allows the targeting of just eukaryotic mRNA. Using this approach, a survey of forest soils helped characterize the taxonomic diversity and also discovered genes that code for novel eukaryotic Carbohydrate-Active enzymes (Damon et al., 2012). Likewise, the large diversity of active protists in mineral and organic soils were identified using the approach (Geisen et al., 2015). Going forward, metatranscriptomics will be pivotal in characterizing diversity of active soil organisms and functions.

### Human Microbiomes

In the past decade, our understanding of the human microbiome has rapidly expanded thanks to sequencing technologies that made possible the description of human gut microbial diversity across large human cohorts (Arumugam et al., 2011; Human Microbiome Project, 2012). Although past studies have primarily focused on describing the taxonomic composition of microbial communities and their functional potential, many studies are now also using metatranscriptomics to better understand the interactions among microbes and their host (Pérez-Losada et al., 2015), to identify active pathways of importance (Franzosa et al., 2014), and how expressed functions may impact disease progression (Nowicki et al., 2018) and severity (Schirmer et al., 2018). A longitudinal study of Inflammatory Bowel Disease (IBD) showed that two organisms *Alistipes putredinis* and *Bacteroides vulgatus* were the sole contributors to the expression of methylerythritol phosphate pathway at different time points. Interestingly, expression by specific organisms correlated with disease severity as *A. putredinis* showed negative and *B. vulgatus* showed a positive correlation (Schirmer et al., 2018). With further advancements in sequencing technologies, laboratory protocols and chemistry, and tailored bioinformatic analysis methods, metatranscriptomics promises to become an integral tool to investigate microbiomes in humans.

### Additional Animal-Microbe Interactions

Metatranscriptomic approaches have also been adapted to better understand the microbiomes of other animals, such as cattles (Mann et al., 2018; Sollinger et al., 2018; Li et al., 2019), squirrels (Hatton et al., 2017), and birds (Marcelino et al., 2019). Many studies in cattle microbiomes are focused on understanding the rumen microbiota to mitigate the release of potent greenhouse gas methane from livestock and increase feed efficiency. Through the use of metatranscriptomics, studies have linked microbes in the rumen to pertinent activities such as methane emission and the degradation of complex plant polysaccharides. For example, Sollinger et al. (2018) found *Prevotella* of *Bacteroidetes* and multiple members of *Firmicutes* were actively involved in the degradation of complex saccharides.

### Plant-Microbe Interactions

Metatranscriptomics has been applied to many plant-microbe interactions studies as it is able to characterize members of a microbiome that are responsible for specific functions and elucidate genes that drive the relationship of the microbiome with its host. Metatranscriptomic sequencing of all community members from roots of the willow plant *Salix purpurea* cv. Fish“ Creek” grown in soil contaminated with petroleum hydrocarbons revealed that the bacterial symbiont *Enterobacteriaceae* was responsible for the degradation of hydrocarbons from among a wide range of active microbes (Gonzalez et al., 2018). The approach is also well suited to detect changes in the microbial community that would have been missed by traditional PCR methods as shown in a study where an increase in diversity of non-fungal eukaryotes was detected in *sad1* mutant of oat plants when compared to its wild type (Turner et al., 2013). The methodology also helped to identify genes that are responsible for the mutualistic relationship of the Seagrass plant with its microbiome members (Crump et al., 2018) and to describe the active microbial communities and pathways in mature ripe fruits (Saminathan et al., 2018). Another example of an attempt to understand mechanisms behind the suppressive and non-suppressive *Rhizoctonia solani* fungal infection in wheat plants revealed a set of genes associated with suppression and non-suppression phenotypes, providing molecular targets for improved agricultural productivity (Hayden et al., 2018).

## Bioinformatic Analysis of Metatranscriptomic Sequencing Data

Because of microbiome complexity, high throughput sequencing in the form of short read data usually generated from Illumina sequencing technology has been most often applied for metatranscriptome studies, particularly when multiple samples and deep coverage are required, such as in differential gene expression studies. Since most information about samples are unknown *a priori*, such as its microbial composition, relative abundance of community membership, genome sizes, and relative expression within and among genomes, it is not trivial to find right experimental parameters such as depth of sequencing for metatranscriptomics. While long read sequencing can produce full or near full-length mRNAs which can help discriminate among different isoforms (Pollard et al., 2018), and provide longer stretches of sequence for similarity searches, the various long read technologies currently only play a supporting role and are not actively being used alone for metatranscriptome studies. Here, we focus on available tools and workflows for metatranscriptome data processing and analysis, which focus on short read data.

### Preprocessing

Similar to other NGS datasets, one of the first steps in processing RNASeq data is to do Quality Control (QC) and remove or trim spurious/erroneous reads to minimize errors. One of the many dozens of available QC tools, such as FastQC (Andrews, 2010), FaQCs (Lo and Chain, 2014), fastp (Chen et al., 2018), and Trimmomatic (Bolger et al., 2014), can be used for short reads derived from Illumina sequencers.

One of the important steps that should be taken into consideration is physical removal or depletion of the highly abundant ribosomal RNA (rRNA) transcripts from the samples, as they often constitute upward of 90% of all data if not removed and do not contribute towards most downstream analyses, such as finding differentially expressed genes or pathway characterization. These rRNAs are often removed using molecular approaches prior to sequencing but their dominance in samples results in some amount of rRNA still being sequenced. Post sequencing, rRNAs can be identified for removal from downstream analyses using tools like SortMeRNA (Kopylova et al., 2012) and barrnap (Seemann, 2014).

There are also cases where one would want to remove a target organism from analysis, such as human reads from human microbiome samples. These reads can be removed using traditional read mapping methods that tags and removes reads that map to human genome (Li et al., 2017), or using faster alignment free methods such as Best Match Tagger (BMTagger) (Rotmistrovsky and Agarwala, 2011) that search for human-specific *k-*mers in reads.

### *De Novo* Assembly

Preprocessed, high-quality reads can now be assembled into putative transcripts using *de novo* assemblers. Given that most microbiomes are not adequately characterized with reference genomes, *de novo* assemblers provide a reference scaffold representing longer, expressed genome segments that can provide a reference set of genes. This provides users the ability to find homologs in a more straightforward fashion, establish taxonomic origin, and serve as a reference for mapping against for expression analysis. Metagenomic assemblers such as MEGAHIT (Li et al., 2015), IDBA-UD (Peng et al., 2012) and metaSPAdes (Nurk et al., 2017) have been designed to tackle complex metagenomes that may share some sequence similarity in highly conserved regions but may vary greatly in terms of relative abundance within the microbiome, and may also harbor population (strain-level) variation. However, the effectiveness of these assemblers in reconstructing transcripts that have their own peculiarities such as introns/exons, different isoforms, and shorter non-coding RNAs (ncRNA), have been seldomly tested, so, it is with caution that one should use metagenomic assemblers on metatranscriptome datasets.

Assemblers such as Trans-ABySS (Robertson et al., 2010), Trinity (Grabherr et al., 2011), BinPacker (Liu et al., 2016), Oases (Schulz et al., 2012), SOAPdenovo-Trans (Xie et al., 2014), IDBA-Tran (Peng et al., 2013), and rnaSPAdes (Bushmanova et al., 2019) attempt to account for the issues in transcriptome sequencing, but were originally designed to assemble transcripts from a single organism. Despite their design towards transcriptomic and not metatranscriptomic datasets, comparisons among some assemblers showed that in general, the tested assemblers Oases, Trinity, Metavelvet, all improved the number of annotated genes from the resulting contigs, with the Trinity assembler outperforming the others (Celaj et al., 2014).

IDBA-MT (Leung et al., 2013), IDBA-MTP (Leung et al., 2014), and Transcript Assembly Graph (TAG) (Ye and Tang, 2016) are *de novo* assemblers that are designed specifically for metatranscriptomes and take into account the unique features of both transcripts and the complex nature of microbial communities. IDBA-MT is built upon IDBA-UD and uses multiple *k* values in a de Bruijn graph while accounting for features associated with mRNAs like uneven sequencing depth and common repeat patterns across different mRNAs, thereby lowering the rate of mis assemblies. Likewise, IDBA-MTP was derived from IDBA-MT to be able to assemble lowly expressed mRNAs. It uses the information of known protein sequences to guide the assembly by starting with smaller *k*-values to construct mRNA sequences which are then included based on their similarity with a known set of proteins. TAG is a comparatively recent assembler that also uses a de Bruijn graph, but to assemble the corresponding metagenome, which is then used as a reference to map the transcriptome reads and reconstruct mRNA sequences by traversing the metagenome assembly graph with mapped transcriptome reads. Since it assumes genes are contiguous (without splicing), this particular tool is ineffective to use in microbiomes that also contain eukaryotes. Furthermore, there is an implicit assumption that the metagenome represents sufficient breadth of the community that all expressed genes can be mapped to the metagenome.

The current state of *de novo* assembly for metatranscriptomic datasets is still in its very early stages. Only a handful of tools have been specifically developed for metatranscriptomics, but their efficacy on diverse datasets has not been tested and their hardware, or memory requirements across an array of community complexities and data volume, have also not been rigorously established.

### Transcript Taxonomy

Similar to the taxonomic profiling that is frequently performed with shotgun metagenomic data, one can use the same suite of tools to perform read- or contig-based taxonomic assignments in order to understand what organisms are actively expressing RNA. A separate and distinct method is to focus solely on rRNAs to assess active members of a community, though as mentioned above, these are frequently removed (both in the wet-lab protocols as well as in preprocessing of the raw data).

Read-based taxonomy classification tools such as Kraken (Wood and Salzberg, 2014), GOTTCHA (Freitas et al., 2015), MetaPhlan2 (Truong et al., 2015), etc. can be used for metatranscriptomes (Neves et al., 2017). Because these tools work on short reads and are based on nucleotide matches, their effective use is limited to microbiomes whose members have close neighbors in existing sequence databases. Reads that have been assembled into longer contigs and possibly full-length transcripts can be used by a number of tools, such as centrifuge (Kim et al., 2016a) and Kraken 2 (Wood and Salzberg, 2014), to potentially identify a greater proportion of the sequenced community members.

Taxonomic assignments using reads or predicted coding regions have a large number of limitations, including the algorithms necessary to process large volumes of data or accommodate short sequences, and the paucity of references in the reference databases. Compounding such issues, is the fact that most bioinformatics tools only utilize a subset of available genomes or focus on certain organisms. For example, many tools do not have eukaryotes as part of their databases. There have been some recent efforts with new tools and improvements in existing tools, to include eukaryotic genomes within their databases, such as MetaPhlan2 (Truong et al., 2015) and kaiju (Menzel et al., 2016), but their efficacy in classifying eukaryotes is unknown. Furthermore, it is often difficult to discern low abundance hits from false positive hits, which is an innate problem with microbiome studies. Our general lack of knowledge on overall microbial diversity and in any biological system under study can also limit the utility of taxonomy classification tools.

### Functional Annotation

One of the main goals of metatranscriptomics is to assess the functional activity of a microbiome. Since the expressed transcripts represent a proxy to the actual phenotype, characterizing the function of transcripts is a fundamental task for metatranscriptomics. Functional annotation can be conducted using either reads or assembled contigs. Read based functional profilers such as MetaCLADE (Ugarte et al., 2018), HMM-GRASPx (Zhong et al., 2016), and UProC (Meinicke, 2015) use tool-specific databases and require predicted open reading frames as input, from other tools like FragGeneScan (Rho et al., 2010). MetaCLADE is one of the latest tools and uses a database that consists of 2 million probabilistic models derived from 15,000 Pfam domains, thus hundreds of models representing any single domain, to encompass the diversity of each domain across the tree of life. A search against this database results in large numbers of hits per read which are then filtered based on redundancy, probability and bit-scores (Ugarte et al., 2018).

Alternatively, annotation of genes can be performed from assembled contigs. Annotation of assembled transcripts proceeds similar to the annotation of genomes and metagenomes. Gene finding using programs like Prodigal (Hyatt et al., 2010) and FragGeneScan (Rho et al., 2010) is followed by functional assignment based on similarity searches using tools such as DIAMOND (Buchfink et al., 2015) to search against functional databases like KEGG (Kanehisa and Goto, 2000), NCBI RefSeq (O’leary et al., 2016), UniProt (Uniprot, 2019) etc. Other tools, pipelines and platforms encompass an array of bioinformatics utilities (including gene finding and annotation), such as Prokka (Seemann, 2014), EDGE Bioinformatics (Li et al., 2017), and MG-RAST (Wilke et al., 2016), which combine a number of similarity searches against various databases, or can even couple assembly, gene calling, and annotation *via* similarity searches. Once annotations are performed, enzymatic functions may also be mapped to known metabolic pathways, using tools like MinPath (Ye and Doak, 2009) or iPath (Yamada et al., 2011).

### Differential Expression Analyses

Beyond the simple description of who are the active members and what genes are being expressed at a single time point, are studies of differential gene expression, where metatranscriptomics can be used to compare differing conditions and environmental parameters and their effect on community function or to observe community dynamics over time. There are many tools originally developed for use with single genomes that can be leveraged for metatranscriptomic differential gene expression studies. These tools require as input abundance data per gene (or transcript) and per sample (representing expression under a specific condition or a specific time point). Abundance can be attained in a number of ways, but typically involves some form of read alignment/mapping to a reference genome, a reference assembly or a reference gene set. EdgeR (Robinson et al., 2010), DeSeq2 (Love et al., 2014), and limma (Ritchie et al., 2015) are R packages that are frequently used, together with the abundance information, to identify genes that are statistically significantly differentially expressed among a number of samples (i.e., conditions/timepoints). Likewise, tools such as Generally Applicable Gene-Set/Pathway Analysis (GAGE) can be used to identify pathways enriched in one condition over another (Luo et al., 2009). Since, replicating metatranscriptomics samples are not trivial compared to transcriptomic studies with isolate organisms, non-parametric methods as the implementation in NOISeq (Tarazona et al., 2015) should also be considered.

There are peculiarities in metatranscriptomic analyses that makes differential expression analyses rather challenging, mainly as a result of sequencing a large diversity of transcripts (from a wide array of organisms). Problems such as shared genes among closely related organisms and variation in the taxonomic composition of transcripts can result in incorrect assessment of gene expression profiles. A normalization method has been recently proposed that can minimize the influence of taxonomic diversity in the sample by normalizing count data based on taxonomic composition across different samples, but this method is also biased by representation in taxonomic databases (Klingenberg and Meinicke, 2017).

## Available Workflows for Metatranscriptomic Analysis

As alluded to above, the analysis of a metatranscriptome dataset is laden with choices of bioinformatic steps with many options for tools for any given step. Which steps and tools should be selected are often dictated by the goals of the experiment, the details of which can grow in complexity based on the specifics of the study. However, there do exist bioinformatic workflows that aim to streamline some of this complexity by connecting multiple individual tools into a workflow that can take raw sequencing reads, and process them providing data files that represent the outputs results characterizing taxonomic identities, functional genes, and/or differentially expressed transcripts. Here we summarize eight of the available workflows, namely MetaTrans (Martinez et al., 2016), COMAN (Ni et al., 2016), FMAP (Kim et al., 2016b), SAMSA2 (Westreich et al., 2018), HUMAnN2 (Franzosa et al., 2018), SqueezeMeta (Tamames and Puente-Sánchez, 2018), IMP (Narayanasamy et al., 2016), and MOSCA (Sequeira et al., 2019). We compare the types of analyses these workflows are capable of performing, which dictates what types of biological questions may be addressed using them. Details of these eight workflows, their capabilities (e.g. QC, assembly, differential gene expression analysis), and the specific bioinformatics tools that they use, can be found as a summary in **Table 1** and in detail in **Supplementary Table 1**.

**Table 1:** A list of metatranscriptomics pipelines and their capabilities.

		Read based					Assembly based		
		MetaTrans	COMAN	FMAP	SAMSA2	HUMAnN2	SqueezeMeta	IMP	MOSCA
Preprocessing	QC	✓	✓	✓	✓	×	✓	✓	✓
	Removes host reads	×	×	✓	×	×	×	✓	×
	Removes rRNA	✓	✓	×	✓	×	✓	✓	✓
*de novo* Assembly		×	×	×	×	×	✓	✓	✓
Binning		×	×	×	×	×	✓	✓	×
Taxonomic Profiling	Reads	✓	✓	×	✓	✓	×	×	×
	Contigs	×	×	×	×	×	✓	✓	✓
Functional Annotation	Reads	✓	✓	✓	✓	✓	×	×	×
	Contigs	×	×	×	×	×	✓	✓	✓
Pathway Analysis		✓	✓	✓	×	✓	✓	✓	×
Requires Metagenomes		×	×	×	×	×	×	✓	×
Summary Report		×	×	×	×	×	×	✓	×
Web Interface		×	✓	×	×	×	×	×	×
Multiple Sample Comparisons		✓	✓	✓	✓	✓	✓	×	✓
Differential Expression		✓	✓	✓	✓	×	×	×	✓
Docker		×	×	×	×	✓	×	✓	✓
Conda		×	×	×	×	✓	×	✓	×
Long Read Support		×	×	×	×	×	✓	×	×
Public Code Repository		✓	×	✓	✓	✓	✓	✓	✓

Almost all eight workflows include a form of preprocessing or quality control of raw data, with the exception of HUMAnN2. All the other workflows, aside from FMAP, include as part of this process the removal of reads matching rRNA prior to other analyses. However, FMAP and IMP allows for the targeted removal of host sequences. After the preprocessing step, all workflows essentially take one of two different approaches, either directly using the reads to perform further analyses, or first performing an assembly and annotation, and then using the annotated genes from that assembly for further analyses (**Supplementary Table 1**). MetaTrans, COMAN, FMAP, SAMSA2, HUMAnN2 all use a read-based approach, while SqueezeMeta, IMP, and MOSCA assemble reads into transcripts before further analyses are performed.

Among all read based workflows, MetaTrans is the only one that first detects putative genes prior to further analyses. All other workflows directly use the filtered reads for similarity searches against taxonomic and functional databases. MetaTrans is also unique in that it utilizes the rRNA sequences that were sequestered in previous step for taxonomic profile analysis. FMAP does not perform taxonomy profiling; and all other workflows use the processed reads to query against a reference database. For these workflows, there are however major differences in how each workflow determines the taxonomy profile. COMAN and SAMSA2 perform their read-based searches in a protein space using DIAMOND, albeit using different reference databases, while HUMANn2 uses MetaPhlan2, which performs searches in nucleotide space. While amino acid based searches allow the detection of organisms distantly related to those in the reference database, they are prone to false discovery. In contrast, nucleotide searches are more specific but are unable to identify sequences insufficiently conserved.

For functional characterization using reads, all five read-based workflows use different algorithms to search for functional similarity using different databases. Only MetaTrans performs these searches in nucleotide space, while all other workflows use read-based predicted peptides as queries. All of the available workflows, aside from SAMSA2, also map predicted proteins onto known pathway maps. Analyses of functional profiles of metatranscriptomes using one of these workflows should be carefully interpreted based on how functions are assigned. For example, functional assignments using searches in nucleotide space, especially for proteins coding genes are likely to be less effective if no near neighbors exist in the reference databases.

In comparison to read-based analyses, assembly-based workflows harbor an extra analytical step, where all the reads are first assembled into larger contigs, which can help reduce the size of the data that needs to be processed for further analyses and increases the contiguous length of the expressed transcripts allowing for more accurate searches. All three of the assembly-based workflows provide multiple assembly tools to choose from, however, IMP has an input requirement, a metagenome dataset that corresponds to the same (or similar) sample as the metatranscriptome. The metagenomic data is used together with the metatranscriptome data for co-assembly. The value of combining metagenome and metatranscriptome dataset is that the assembly becomes more representative of the actual community. IMP uses a corresponding metagenome dataset to create better references through iterative assembly of metagenomes and metatranscriptomes. Both SqueezeMeta and IMP can, in addition, perform post-assembly contig-binning to help group together contigs (i.e. transcripts) into bins representing the same taxon (i.e. genes expressed from the same genome/species). In all three assembly-based workflows, the final contigs are processed to find genes, to perform taxonomy classification with those genes, and to assign them a function.

While all workflows use the identified genes as a query against a reference protein database for taxonomic classification purposes, each workflow uses a different strategy. The reference databases used are different (e.g. Uniprot vs NR), and each workflow assigns taxonomy using different algorithms and scoring thresholds (i.e. last common ancestor vs best hit). The SqueezeMeta workflow also uses the rRNA reads that were extracted during the preprocessing step to provide an additional community profile. One major drawback that is common among several workflows is the implementation of an unorthodox approach of assigning taxonomy by searching against databases that are designed for functional characterization.

For functional annotation, the IMP workflow simply uses the output of the Prokka pipeline that was used for gene identification and annotation. The MOSCA workflow uses the output of the taxonomic search against Uniprot and assigns functional annotation based on best hit, while SqueezeMeta performs additional Hidden Markov Model searches against several protein family databases. The SqueezeMeta and IMP workflows also provide pathway analysis based on the annotated functions.

Because one of the primary goals of metatranscriptome analyses is to obtain a relative quantification of gene expression, all read-based and assembly-based workflows provide some form of per gene coverage and/or abundance metric (e.g. raw count per gene, or number of reads per kb per million reads sequenced). These abundance values can be used with additional tools to compare relative gene expression between growth conditions or during time-course experiments, the purpose of which is often to help understand what genes and pathways may be important for a particular phenotype under study. For these types of studies, replicate experiments are often required to obtain statistically significant results, thus the relative gene abundance comparisons is often a comparison among many different samples that include several biological replicates. MetaTrans, FMAP, COMAN, and MOSCA innately provide such a comparative capability within their workflows, can process several datasets and generate a list of genes that are found to be statistically significantly differentially expressed between different conditions (or time points). SAMSA2 also allows differential gene expression analysis but requires individual sample processing followed by the use of an additional command line utility provided as part of the package.

All workflows, with the exception of COMAN, provide a code repository and is invoked using Command Line Interface. COMAN provides a web server interface. The availability of multiple workflows enables users to choose the one that is the most appropriate for analyzing their metatranscriptome. While users should ideally select workflows based on capability/functionality and quality of the algorithms/tools used, additional considerations may include the computational resource requirements, which vary among workflows, and the frequency of maintenance or active development of the source code, which can undergo frequent modifications as new advances, tools, or methods continue to be developed. Both **Table 1** and **Supplementary Table 1** are compilations of these available workflows and can be used as a potential guide to choose a workflow based on factors that are important to address any researcher’s question(s). For example, if differential expression analysis is the goal of a study, the list of workflows to choose from is limited to five.

## Metatranscriptomics—a Future Full of Promises and Challenges

As alluded to above, it is clear that the next generation sequencing revolution that has taken place in the study of genomes and metagenomes has been successfully adapted to the study of gene expression with ”RNAseq,” and further, to the study of complex biological system dynamics with metatranscriptomics. This new field has seen a rapid increase in the number of metatranscriptomic projects, most of which represent differential gene expression studies whose goals include obtaining insight into the active members, genes, and pathways within a microbiome. That goal, however, is plagued by the lack of adequate reference genomes, which can result in a suboptimal fraction of reads from any dataset from being functionally or taxonomically characterized. It is for this reason that efforts remain to assemble metatranscriptomic data (together with metagenomic data from the same, or similar sample, if available).

While metatranscriptomic data deposited into public repositories enable future big data analytics and global meta-analyses for discovery of important genes, pathways, and organisms, a prerequisite is the concomitant availability of sample and experimental metadata that help define the context of these complex datasets. While over time, a larger fraction of available metatranscriptomes has been deposited with some metadata (**Figure 1**), to realize the full potential of metatranscriptomic meta-analyses, or for any form of metatranscriptome reanalysis, the deposition of adequate sample metadata should become an important focus of future efforts, together with standardization of vocabulary for metadata descriptors. Several grass-roots efforts among the larger scientific community such as Minimum Information about any Sequence or MIxS (Yilmaz et al., 2011) will be needed if we hope to set a series of standards for inclusion of sufficiently detailed metadata when depositing metatranscriptomic (or any omics) datasets that would allow such all-inclusive analyses.

Because of the broad dynamic range of both microbiome membership relative abundance and of gene expression within any given organism, metatranscriptomics requires a very large number of data points (i.e. reads). Therefore, high throughput short read technologies dominate this area, however the rise of long read technologies holds great promise when throughput (per dollar) improves. Longer reads will be able to help with all aspects of analysis (assembly, taxonomy determination, functional analysis), and will additionally provide better resolution of transcript isoforms, polycistronic operons, and different genes with high similarity.

While today’s studies are primarily performed with a single short read technology (i.e. Illumina), there exist a large number of analytical tools to aid in all aspects of data analysis. In this review, we highlight some of the major methods of analyzing metatranscriptomics data, some of the specific bioinformatics tools used to accomplish these analyses, and some more complex metatranscriptomic workflows that combine a number of these tools to address several biological questions with minimal input or effort from the users. Each of the workflows uses either a read-based or an assembly-based approach towards taxonomic and/or functional analysis of organisms and genes expressed within a community, and their relative abundances. Some of the workflows can even proceed all the way to performing differential gene expression analysis among various input samples. While the workflows share a number of similarities, the tools used differ, and it is not clear which workflow, or bioinformatics tool, may be best under any given scenario. Thus, one additional area that beckons for more research is the benchmarking of the performance and accuracy of bioinformatics tools and pipelines with metatranscriptomic data. The complexity of real microbiomes and our incomplete knowledge of the organisms (or genome sequences) present within them have been great challenges in trying to perform such benchmarking experiments. While we have yet to create tools that are truly able to mimic real sequencing datasets, methods that generate simulated sequencing data from known genomes may be used to create a range of simulated metatranscriptome datasets that can in turn be used to test the behavior of bioinformatics tools and parameter settings. Past efforts have focused on *ad hoc* metrics to evaluate performance using real samples and sequencing data. To make matters more complex, further advancements in sequencing technologies will continue to push the development of new tools and workflows. An accepted framework for benchmarking new tools would help the field progress, and possibly coalesce towards accurate and appropriate workflows. Despite some of the issues with metatranscriptomics as a method, the continued development of new tools and algorithms for analyzing metatranscriptomic data coupled with our increasing understanding of the challenges presented by such datasets, it is clear that the next generation of metatranscriptomics tools hold great promise in facilitating our understanding of the biologically active fraction of microbiomes, and the relevant pathways involved.

## Author Contributions

PC and MS wrote the manuscript with inputs from CL. All authors read and approved the manuscript.

## Funding

This work was supported by the US Defense Threat Reduction Agency (grants CB10152, R-00480-16-0, and CB10623 to PC) and by the U.S Department of Energy, Office of Science, Biological and Environmental Research Division, under award numbers LANLF59T and LANLF59C to PC.” (KP1601010 and 4000150817 877 to PC).

## Conflict of Interest Statement

The authors declare that the research was conducted in the absence of any commercial or financial relationships that could be construed as a potential conflict of interest.
